# Tildrakizumab and Quality of Life: Deep Dive into the Impact of Psoriasis and Treatment on Different Domains—Should Psychosocial Life Impairment Be Considered a Comorbidity?

**DOI:** 10.3390/jcm14010223

**Published:** 2025-01-02

**Authors:** Gaetano Licata, Eugenia Veronica Di Brizzi, Franco Castelli, Giorgia Giuffrida, Elena Stroppiana, Annunziata Dattola, Antonio Giovanni Richetta, Elena De Col, Rossana Peila, Niccolò Siliquini, Carmen Solaroli, Roberta Zanetta, Emilia Cerulli, Giovanna Galdo, Domenico Giordano, Elisa Faure, Valeria Papaianni, Ginevra Pertusi, Maria Teresa Uzzauto, Francesco Loconsole, Leonardo Zichichi

**Affiliations:** 1 Dermatology Unit, San Antonio Abate Hospital, 91016 Trapani, Italy; l.zichichi@gmail.com; 2 Dermatology Unit, Department of Mental and Physical Health and Preventive Medicine, University of Campania Luigi Vanvitelli, 80131 Naples, Italy; eugeniaveronica.dibrizzi@gmail.com; 3 Dermatology Unit, Ospedale Koelliker, 10024 Torino, Italy; franco.castelli@hotmail.it (F.C.); carmensolaroli@libero.it (C.S.); 4 Dermatology Unit, A.O.U. Policlinico “G. Rodolico–S. Marco”, 95100 Catania, Italy; giorgiagiuffrida@hotmail.com; 5 Dermatology Unit, A.O. Città della Salute e della Scienza, 10024 Torino, Italy; elena.stroppiana@gmail.com (E.S.); nic.siliquini@gmail.com (N.S.); 6 Dermatology Unit, Department of Clinical Internal, Anesthesiological and Cardiovascular Science, University of La Sapienza, 00042 Rome, Italy; nancydattola@gmail.com (A.D.); antonio.richetta@uniroma1.it (A.G.R.); 7 Dermatology Unit, Palasalute ASL 1, 18100 Imperia, Italy; elenadecol@libero.it; 8 Dermatology Unit, ASL TO 4, 10081 Castellamonte, Italy; rossanapeila@libero.it; 9 Dermatology Unit, ASL Stresa, 28838 Stresa, Italy; zanettaroberta@yahoo.it; 10 Dermatology Unit, Ospedale S. Maria della Misericordia, 06121 Perugia, Italy; emilia.cerulli@ospedale.perugia.it; 11 Dermatology Unit, Ospedale S. Giuseppe Moscati, 83100 Avellino, Italy; giovannagaldo@libero.it; 12 Dermatology Unit, A.O.U. Sant’Andrea, 00042 Roma, Italy; domenico.giordano1989@gmail.com; 13 Dermatology Unit, Policlinico di Milano Ospedale Maggiore Fondazione IRCCS Ca’, 18001 Granada, Italy; elisa.faure87@gmail.com; 14 Dermatology Unit, A.O.U. P. “G. Martino”, 98121 Messina, Italy; valeriapapaianni82@hotmail.it; 15 Dermatology Unit, P.O. Sant’Andrea, 13100 Vercelli, Italy; ginevra.pertusi@aslvc.piemonte.it; 16 Dermatology Unit, Ospedale Tortora, 84016 Pagani, Italy; mateuzz@tiscali.it; 17 Dermatology Unit, Policlinico di Bari–A.O.U. Consorziale Policlinico, 70100 Bari, Italy; franciscus59@gmail.com

**Keywords:** psoriasis, quality of life, tildrakizumab

## Abstract

**Background/Objectives**: Psoriasis is a chronic inflammatory skin disease that may have a significant impact on patients’ quality of life. Alongside clinical scores, treatment goals include improvements in patients’ quality of life, divided into its social, working and psychosocial life aspects. Indeed, psychological impairment should always be considered in the management of moderate-to-severe psoriasis. Tildrakizumab, an anti-IL-23, is approved for the management of moderate-to-severe psoriasis. Both clinical trials and real-life studies show its efficacy and safety; however, no studies have evaluated how tildrakizumab may improve different domains of quality of life, including physical, psychological, and social aspects of patients’ quality of life. The objective was to evaluate the effectiveness of tildrakizumab in the management of moderate-to-severe psoriasis, focusing on the impact on all domains of patients’ quality of life. **Methods**: A 28-week multicenter, real-life, retrospective study was performed enrolling patients affected by moderate-to-severe psoriasis undergoing treatment with tildrakizumab. PASI and DLQI were evaluated at each follow-up (W16, W28). A sub-analysis of each DLQI question evaluated different domains of quality of life, including physical, psychological, and social aspects of patients’ quality-of-life. **Results**: A total of 62 patients were enrolled. At week 28, 97.1%, 85.7%, and 54.3% of patients achieved PASI75, PASI90, and PASI100, respectively. DLQI showed a significant reduction from baseline (20.3 ± 5.5) to week 28 (0.9 ± 2.2, *p* < 0.0001), with up to 82.9% achieving DLQI < 1. Sub-analysis of each question (Q1–Q10) showed a reduction in the value of each answer from baseline to week 28. **Conclusions**: The results confirm tildrakizumab as an effective and safe treatment in real life, positively affecting all domains of quality of life, with significant impact already appreciable at week 16 of follow-up.

## 1. Introduction

Psoriasis is a chronic inflammatory skin disease in which systemic inflammation has been well recognized [1]. Particularly in its moderate to severe forms, as well as in milder forms affecting sensitive areas, psoriasis may have a significant and negative impact on patients’ quality of life [2]. Psychology and social stress can be combined with other factors such as vaccinations or infections; the stress-induced rise in leukocyte trafficking to immune activation sites triggered by, for example, vaccinations plays a important role in exacerbating autoimmune diseases, such as psoriasis [3,4,5]. The introduction of biologics in daily clinical practice deeply changed the management of psoriasis [6]. Indeed, over the years even the goals of treatment changed due to better efficacy and safety profiles of new biological agents. Treatment goals moved from a 50% reduction in basal Psoriasis Area Severity Index (PASI50), to PASI 75, PASI90, and PASI100, which means complete skin clearance [6,7]. Moreover, along with objective clinical score as PASI and body surface area (BSA), the treatment goals also include improvements in patients’ quality of life, divided into its social, working and psychosocial life aspects, the so-called “patient’s wellbeing” [8]. Increased pro-inflammatory mediators are present in both psoriasis and depression, suggesting a potential pathophysiological link between the diseases. Anti-interleukin (IL)-23 represents the latest class of biologics approved for the management of psoriasis. This class of drugs has showed to be among the safest and most effective treatments for the management of moderate to severe forms of psoriasis [9]. Tildrakizumab, a humanized IgG1 monoclonal antibody targeting IL-23 p19, is approved for the management of moderate to severe forms of plaque psoriasis [10]. As with other anti-IL-23s, several studies have already showed its efficacy and safety in both randomized clinical trials and real-life studies; however, only a few data have been reported about its impact on patients’ quality of life in daily clinical practice [11,12,13,14,15,16,17,18,19,20]. In particular, no study evaluated how psoriasis may affect the different domains of quality of life, including physical, psychological, and social aspects of patients’ quality of life. Herein, we report the results of a multicentric retrospective study evaluating the effectiveness of tildrakizumab in the management of moderate to severe psoriasis, focusing on the impact that this treatment has on all domains of patients’ quality of life.

## 2. Materials and Methods

A 28-week multicenter, real-life, retrospective study was performed enrolling patients affected by moderate-to-severe plaque psoriasis undergoing treatment with tildrakizumab attending the Psoriasis Center at 19 Italian centers. Inclusion criteria were diagnosis of moderate-to-severe plaque psoriasis for at least 6 months assessed by a dermatologist, patients receiving tildrakizumab, and absence of contraindications to the administration of the drug.

Exclusion criteria were: clinically important active infections; pregnancy; breastfeeding or women of childbearing age who were unwilling to use appropriate contraceptive methods; medical problems that in the investigator’s opinion would put the patient at risk of adverse events; history of allergy to any component of tildrakizumab.

Tildrakizumab was administered at the labelled dosage (100 mg or 200 mg based on patients’ features and clinical decision) by subcutaneous injection at weeks 0 and 4.

Patients who were already receiving biologic therapy were required to undergo a sufficient wash-out period from their current medication prior to initiating tildrakizumab therapy.

At baseline, demographic (age, sex) and clinical data [psoriasis duration, presence of psoriatic arthritis (PsA), involvement of difficult-to-treat areas (scalp, nails, palms or soles, genitals), comorbidities, previous conventional and biologic treatments for psoriasis, psoriasis severity by using Psoriasis Activity Severity Index (PASI) and impact on quality of life using Dermatology Life Quality Index (DLQI)] were collected. Patients with a DLQI < 10 at baseline were excluded to enroll only patients reporting a high impact on their quality of life, to evaluate how tildrakizumab may impact this group of patients.

PASI and DLQI were evaluated at each follow-up visit [week (W)16, W28].

A sub-analysis of each question of the DLQI was performed (Table 1).

A clinical outcome below PASI75 after 28 weeks was labeled as primary lack of effectiveness, while secondary ineffectiveness was considered to be a lack of PASI75 response after an initial positive clinical outcome after 28 weeks of treatment.

The present study was conducted respecting the Declaration of Helsinki, and all patients signed an informed consent before starting the study.

Statistical analysis

Statistical evaluation was carried out to assess the significance of the results. Clinical and demographic data were shown using descriptive statistics, presenting as mean ± standard deviation in case of continuous variables and using number and proportion for categorical ones.

Moreover, Student’s *t*-test was used to assess the statistical significance of clinical improvement evaluated at the different timepoints as compared with baseline.

Statistical analysis using GraphPad Prism 8.0 (GraphPad Software Inc., La Jolla, CA, USA) was used to evaluate the statistical significance of clinical response, considering statistically significant a *p*-value < 0.05.

## 3. Results

A total of 81 patients (49 male, 60.5%; mean age: 52.6 ± 15.1 years) were screened. Among these, 62 subjects (34 male, 54.8%; mean age: 52.0 ± 15.9 years, mean psoriasis duration: 19.8 ± 14.2 years) with a DLQI ≥ 10 at baseline (DLQI: 20.3 ± 5.5) were enrolled.

Tildrakizumab was scheduled at dosages of 100 mg or 200 mg in 47 (75.8%) and 15 (24.2%) patients, respectively, without dosage adjustment during the follow-up period.

Twenty-nine patients (46.8%) were affected by at least one comorbidity, with hypertension (n = 15, 24.2%), dyslipidemia (n = 15, 24.2%), and diabetes (n = 9, 14.5%) as the most common.

Moreover, 36 (58.1%) patients were bio-naïve. Among the bio-experienced patients (n = 26, 41.9%), adalimumab was the most common biologic administered (n = 9, 14.5%) followed by secukinumab (n = 7, 11.3%) etanercept (n = 4, 6.4%) and infliximab (n = 4, 6.4%). As regards difficult-to-treat area involvement, scalp, genitals, nails and palms or soles were involved in 37 (59.7%), 25 (40.3%), 11 (17.7%), and 13 (21.0%) subjects, respectively. Finally, 7 (11.3%) patients had concomitant PsA.

Clinical and demographic features are summarized in Table 2 and Table 3.

At baseline, psoriasis assessment showed a mean PASI of 15.6 ± 6.5.

A total of 55 (88.7%) and 35 (56.5%) patients reached week 16 and week 28 follow-up, respectively.

Statistically significant improvements in PASI were observed at week 16 (PASI: 3.0 ± 3.9, *p* < 0.0001) and week 28 (PASI: 1.2 ± 2.2, *p* < 0.0001) compared with baseline.

In total, 41/55 (74.5%) and 34/35 (97.1%) reached PASI75 at weeks 16 and 28, respectively. Moreover, among the 55 subjects reaching week 16 of follow-up, PASI90/100 were achieved by 28 (50.9%) and 19 (34.5%) patients, while 30 (85.7%) and 19 (54.3%) of the patients achieving week 28 reached PASI90 and PASI100 response at this timepoint, respectively (Table 3).

**Figure 1:**
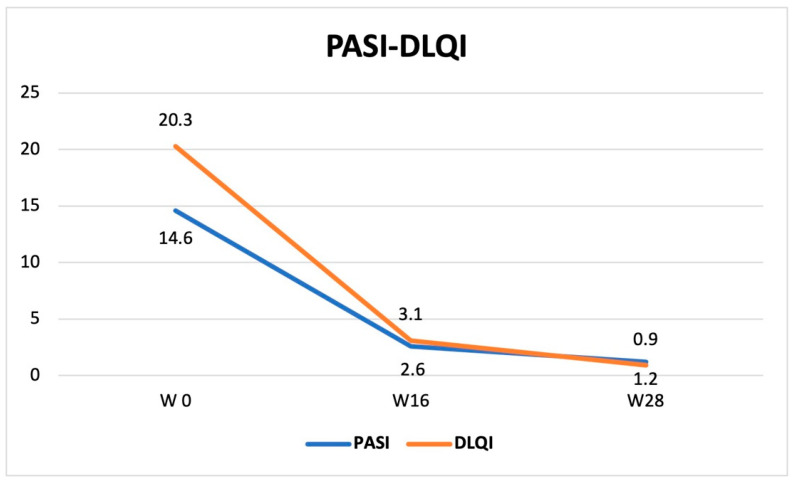
PASI and DLQI improvement from baseline to week 16 and week 28.

Similarly, DLQI showed a significant reduction from baseline (20.3 ± 5.5) to weeks 16 (3.1 ± 3.8, *p* < 0.0001) and 28 (0.9 ± 2.2, *p* < 0.0001). In particular, 24/55 (43.6%) and 29/35 (82.9%) achieved DLQI < 1 at these timepoints, respectively (Figure 1).

**Figure 2:**
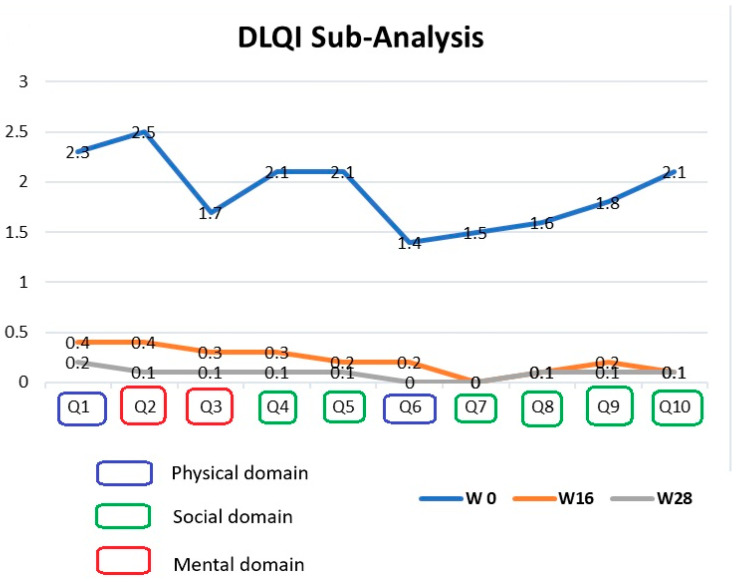
Sub-analysis of each DLQI question (Q1–Q10) from baseline to week 16 and week 28.

Alongside the average DLQI score, a subanalysis of each DLQI question was performed considering the different domains analyzed by the DLQI questionnaire. In particular, Q1 and Q6 were used to assess the physical domain, Q2 and Q3 the mental domain, and Q4, Q5, and Q7–Q10 the social domain. Sub-analysis of each question (Q1–Q10) showed a reduction for the value of each answer from baseline to week 28 (Q1: from 2.3 to 0.2; Q2: from 2.5 to 0.1; Q3: from 1.7 to 0.1; Q4: from 2.1 to 0.1; Q5: from 2.1 to 0.1; Q6: from 1.4 to 0; Q7: from 1.3 to 0; Q8: from 1.6 to 0.1; Q9: from 1.5 to 0.1; Q10: from 1.9 to 0.1), highlighting a positive impact of tildrakizumab on all analyzed domains (Figure 2).

No AEs or treatment discontinuations for safety issues or ineffectiveness were reported in our study.

## 4. Discussion

Psoriasis is a chronic inflammatory disease which may have a significantly negative effect on patients’ quality of life [21]. Indeed, the severity of manifestation as well as the several comorbidities linked to psoriasis may deeply affect the daily lives of patients. Severe clinical manifestations affecting large body surface areas, as well as the involvement of “sensitive areas” such as the face, scalp, palm-plantar region, and genitalia, may result in disordered appearance, social and working issues, and a generally lower quality of life [22]. Indeed, stigmatization and psychological distress are well known features of patients suffering from psoriasis [23]. Furthermore, psychological distress, which may be related to the impact on patients’ quality of life, is described as a potential trigger factor for the recurrence and worsening of psoriasis [24]. In particular, it has been supposed that psychological distress may be associated with changes in the phenotype of circulating lymphocytes, representing a stimulating factor for the activation of the T-helper 1 cell-polarized inflammatory response in psoriatic skin lesions [25]. Being able to reduce the inflammatory response, not merely in the skin but also the systemic inflammation reported in the disease, is associated with an improvement in both objectively examinable symptoms (i.e., skin manifestations and articular symptoms) and the impairment that psoriasis leads on several aspects of patients’ life.

The introduction of biologic treatments in daily clinical practice deeply changed the way moderate to severe psoriasis is managed. Indeed, over the years even treatment goals changed, moving from PASI50 to PASI90 and PASI 100. Along with objective clinical scores, the improvement of patients’ quality of life became as important as PASI, BSA or IGA [26].

Indeed, as with other comorbidities, several studies evaluated the impact of new available drugs on patients’ quality of life, which may also be linked to increased patient compliance with treatments, as well as reduced flares during treatments [9]. Nowadays, patient wellbeing is increasingly considered among the main treatment goals in the management of moderate to severe psoriasis [9]. A nationwide Italian survey, the SHAPE (SHAring Patient Experiences) study, evaluated patients’ perspectives on the impact of psoriasis on their wellbeing [8]. The authors’ results showed that psoriatic patients reported considering physical, mental and social health to be the three core domains associated with their wellbeing, giving the same importance to all of them [9]. The SHAPE study also investigated patients’ perceptions of their ongoing treatment. Interestingly, they found that the expectation of patients from ongoing treatment and their perception of the disease exceeded the reality that they were currently experiencing in terms of their treatment and disease perception [9]. Moreover, another important finding reported by the study was that only about 24% of patients felt that their dermatologist had considered disease-related quality of life, with up to 40% of them reporting feeling that their dermatologist was not giving enough importance to these aspects during treatment and routine visits [9]. These results were in line with literature data reporting that the social and psychological impact of psoriasis may be underestimated by dermatologists [26,27,28,29,30]. These results show that more attention should be given to quality-of-life-related aspects during visits and in treatment choices. Indeed, recently reported data showed that the prioritization of patients’ quality of life in psoriatic patients could be linked to higher treatment compliance, and eventually to better clinical results [28,29]. However, only a poor concordance has been reported between PASI or BSA and DLQI improvement, highlighting the need for new scores and/or questionnaires to evaluate both clinical and psychological aspects during treatment [31]. Thus, these data highlight how improvement in skin manifestations alone is not linked to an improvement in patients’ quality of life, raising questions about the need for follow-up over time, as is usually performed with other well-known comorbidities (i.e., psoriatic arthritis). The lack of measures for assessing the overall well-being of patients with psoriasis is an unmet need in both daily dermatological practice and clinical research. Psoriasis significantly impacts the physical, psychological and social well-being of patients and their family members/partners; hence, beside clinical assessments, the full impact of psoriasis treatment on the patient and surrounding people should not be underestimated [32]. All domains of patients’ lives are frequently affected in different ways, including physical, psychological and social aspects of daily life.

As previously stated, the availability of biologics positively impacted the way psoriasis is managed. Among available biologics, anti-IL-23s represent the latest class available in clinical practice for the treatment of psoriasis. Among this class, Tildrakizumab is a humanized IgG1κ monoclonal antibody targeting the p19 subunit of IL-23, approved for the treatment of patients with moderate-to-severe chronic plaque psoriasis [10]. Several studies evaluated tildrakizumab’s efficacy and safety in the management of psoriasis. The first published trials were reSURFACE 1 and reSURFACE 2 [16]. The reSURFACE 1 study evaluated the efficacy of tildrakizumab 100 mg and tildrakizumab 200 mg versus placebo, while reSURFACE 2 compared the efficacy of tildrakizumab 100 and 200 mg versus etanercept 50 mg and placebo [18]. Both studies reported higher efficacy of tildrakizumab in reaching PASI 90 and PASI 100 responses. Focusing on quality of life, reSURFACE1, after only 12 weeks of treatment, reported achievement of DLQI 0 or 1 in 44% and 42% of tildrakizumab-treated patients (200 mg and 100 mg groups respectively), vs. only 5% of those treated with placebo [18]. These responses were maintained and increased at the week 28 follow-up, where 57% and 52% of patients receiving tildrakizumab 200 mg and 100 mg achieved DLQI 0/1 [18]. Moreover, comparable results were reported in patients who switched from placebo to tildrakizumab after week 12. As regards reSURFACE2 trial, at week 28 DLQI 0 or 1 were achieved in up to 65% and 54% of the tildrakizumab 200 mg and 100 mg treated groups vs. 39% of those receiving etanercept [18]. A post hoc analysis of the reSURFACE studies aimed to evaluate the efficacy of tildrakizumab and the impact on quality of life [30]. In particular, this analysis evaluated tildrakizumab effectiveness in patients with different levels of PASI improvements. Patients enrolled in reSURFACE1 and reSURFACE2 trials treated with tildrakizumab 100 mg or 200 mg were pooled and divided into five groups according to week 28 PASI improvement (PASI 0–49, 50–74, 75–89, 90–99 and 100) [30]. Quality of life was evaluated for each group at baseline, weeks 12, 28, 40 and 52 [30]. For patients in the week 28 PASI < 50, PASI 50–74, PASI 75–89, PASI 90–99 and PASI 100 response cohorts, the rates of DLQI 0/1 achievement at week 28 were 8.3%, 22.0%, 40.9%, 66.3% and 86.5%, respectively, in the 100 mg cohort, and 8.7%, 35.2%, 43.9%, 70.4% and 85.9%, respectively, in the 200 mg cohort, demonstrating that patients with a higher PASI response were also those with a better QoL improvement. Similar results were shown in patients receiving both tildrakizumab 100 mg and 200 mg up to week 52 [30].

The TRIBUTE study, an open-label Phase IV study, aimed to assess the efficacy and impact on health-related quality of life (HRQoL) of tildrakizumab in patients with moderate-to-severe psoriasis in conditions similar to clinical practice. The TRIBUTE study showed a significant improvement in patients’ quality of life, which was even associated with significant positive impacts on work productivity and activity already at week 24 [32]. Furthermore, beyond the DLQI, the study also evaluated the patients’ benefit index (PBI), showing that up to 99% of patients after only 24 weeks of treatments reached a PBI total score equal to or higher than 1 [32].

Here, we reported the results of a multicenter, retrospective study that enrolled patients affected by moderate-to-severe plaque psoriasis undergoing treatment with tildrakizumab. Our study confirmed the high efficacy of tildrakizumab showed in trials and several real-life studies. Indeed, our cohort of patients reported a statistically significant reduction in mean PASI, which reduced from 15.6 ± 6.5 at baseline to 3.0 ± 3.9 (*p* < 0.0001) at week 16, and up to 1.2 ± 2.2 (*p* < 0.0001) at week 28.

Moreover, a high rate of patients reached PASI75, 90 and 100 responses. Specifically at week 16 and week 28, PASI 75 was reached by 74.5% and 97.1% of patients, respectively, PASI 90 by 50.9% and 85.7%, respectively, and PASI 100 by 34.5% and 54.3%, respectively. In line with the PASI trend, mean DLQI showed a significant reduction during the study, reducing from 20.3 ± 5.5 to 3.1 ± 3.8 (*p* < 0.0001), at week 16, and up to 0.9 ± 2.2 (*p* < 0.0001) at week 28. Notably, 43.6% and 82.9% of patients achieved DLQI < 1 at week 16 and week 28, respectively.

Our results confirm the positive and significant role that tildrakizumab treatment has on patients’ quality of life. Interestingly, a sub-analysis of each DLQI question (Q1–Q10) showed a significant reduction for the value of each answer, indirectly highlighting a significant improvement in the impact that psoriasis has on different aspects of patients’ quality of life (Figure 2). In particular, our analysis showed that the psychological and social impairments could be compared to the physical impairment in patients with moderate to severe psoriasis. Specifically, physical (Q1, Q6 of DLQI), psychological (Q2–Q3 of DLQI), and social (Q4, Q5, Q7–Q10 of DLQI) aspects showed at baseline mean values of 1.9, 2.1, and 1.8, respectively, showing how all different aspects of patients’ life are affected by the disease. Particularly, psychological impairment identified by Q2 and Q3 showed an even higher impact than that showed by physical questions of the DLQI (Q1 and Q6), which were comparable to social impairment. This sub-analysis highlighted how social and psychological impairments are not strictly linked to cutaneous symptoms. Interestingly, all mean scores showed a similar decreasing trend between tildrakizumab treatment at week 16 and week 28, reducing to 0.3 and 0.1 for the physical domain, to 0.4 and 0.1 for the psychological domain, and to 0.2 and 0.1 for the social domain. Thus, tildrakizumab was able to positively affect all examined domains during treatment, with significant positive impact already appreciable as early as week 16 of follow-up.

Near these significant results, our study also confirmed the safety of tildrakizumab in real life, with no AEs or treatment discontinuations for safety issues or ineffectiveness reported during the study.

## 5. Conclusions

The impact that psoriasis has on patients’ quality of life may be severe and involve all aspects of patients’ life, ranging from work productivity to social activity, and psychosocial aspects.

Thus, dermatologists should look beyond the skin, regularly investigating these aspects and considering health-related quality of life among the main treatment goals during follow-ups. Our results showed tildrakizumab to be a promising treatment to improve the impact that psoriasis has on patients’ quality of life. In line with the literature, tildrakizumab resulted in a safe and effective treatment for the management of moderate to severe psoriasis. Our data, in line with the literature, show that the impact of psoriasis on daily patients’ life should not be underestimated, needing continuous follow-up as with other diseases linked to psoriasis. These data underline the importance that dermatologists and other physicians managing psoriatic diseases should have in daily practice, raising the question “Should psychosocial impairment be considered a comorbidity?”. Further studies would confirm the importance that management of impairment of quality of life has in the daily management of psoriatic patients, to create a specific protocol and call for action for dermatologists to increase attention to this long-underestimated comorbidity.

## Figures and Tables

**Table 1:** DLQI questions.

DLQI Questions (Scoring 0: Not at All/Not Relevant, 1: A Little, 2: A Lot, 3: Very Much)
Q1: Over the last week, how itchy, sore, painful or stinging has your skin been?
Q2: Over the last week, how embarrassed or self-conscious have you been because of your skin?
Q3: Over the last week, how much has your skin interfered with you going shopping or looking after your home or garden?
Q4: Over the last week, how much has your skin influenced the clothes you wear?
Q5: Over the last week, how much has your skin affected any social or leisure activities?
Q6: Over the last week, how much has your skin made it difficult for you to do any sport?
Q7: Over the last week, has your skin prevented you from working or studying? If “No”, over the last week how much has your skin been a problem at work or studying?
Q8: Over the last week, how much has your skin created problems with your partner or any of your close friends or relatives?
Q9: Over the last week, how much has your skin caused any sexual difficulties?
Q10: Over the last week, how much of a problem has the treatment for your skin been, for example by making your home messy, or by taking up time?

DLQI: Dermatology life quality index.

**Table 2:** Characteristics of the study population.

**Number of Patients**	62
**Sex:**	
Male	34 (54.8%)
Female	28 (45.2%)
**Mean age** (years)	52.0 ± 15.9
**Mean duration of psoriasis** (years)	19.8 ± 14.2
**Psoriatic Arthritis**	7 (11.3%)
**Difficult-to-treat area involvement**	
Scalp	37 (59.7%)
Palms or soles	13 (21.0%)
Genitals	25 (40.3%)
Fingernails	11 (17.7%)
**Patients with at least one comorbidity:**	**29 (46.8%)**
Hypertension	15 (24.2%)
Dyslipidemia	15 (24.2%)
Diabetes	9 (14.5%)
Depression	4 (6.5%)
Cardiopathy	3 (4.8%)
Obesity	2 (3.2%)
Kidney failure	2 (3.2%)
Other comorbidities	9 (14.5%)
**Previous systemic treatments (conventional and small molecules):**	
Methotrexate	17 (27.4%)
Cyclosporine	21 (33.9%)
Phototherapy	10 (16.1%)
Acitretin	8 (12.9%)
Apremilast	3 (4.8%)
Dymethil Fumarate	2 (3.2%)
**Bio-naïve:**	36 (58.1%)
**Bio-experienced:**	
**Anti-TNFα**	**17 (27.4%)**
Adalimumab	9 (14.5%)
Etanercept	4 (6.4%)
Infliximab	4 (6.4%)
Golimumab	0
Certolizumab	0
**Anti-IL12/23**	**3 (4.8%)**
**Anti-IL17**	**10 (16.1%)**
Secukinumab	7 (11.3%)
Ixekizumab	3 (4.8%)
Brodalumab	1 (1.6%)
**Anti-IL23**	**1 (1.6%)**
Risankizumab	1 (1.6%)
Guselkumab	0

IL: Interleukin; TNF: Tumor Necrosis Alpha.

**Table 3:** Psoriasis assessment at baseline, and after tildrakizumab treatment at week 16 and week 28. PASI: Psoriasis Activity Severity Index. DLQI: Dermatology Life Quality Index.

	Baseline	Week 16	Week 28
Mean PASI	15.6 ± 6.5	3.0 ± 3.9	1.2 ± 2.2
Mean DLQI	20.3 ± 5.5	3.1 ± 3.8	0.9 ± 2.2
PASI75	N/A	41/55 (74.5%)	34/35 (97.1%)
PASI90	N/A	28/55 (50.9%)	30/35 (85.7%)
PASI100	N/A	19/55 (34.5%)	19/35 (54.3%)

DLQI: Dermatology life quality index; PASI: Psoriasis area severity index.

## Data Availability

Data that support the results of this study are available from the corresponding author upon reasonable request.

## References

[1] Christophers E., van de Kerkhof P.C.M. (2019). Severity, heterogeneity and systemic inflammation in psoriasis. J. Eur. Acad. Dermatol. Venereol..

[2] Basavaraj K.H., Navya M.A., Rashmi R. (2011). Stress and quality of life in psoriasis: An update. Int. J. Dermatol..

[3] Marasca C., Ruggiero A., Napolitano M., Fabbrocini G., Megna M. (2020). May COVID-19 outbreaks lead to a worsening of skin chronic inflammatory conditions?. Med. Hypotheses.

[4] Karampinis E., Papadopoulou M.-M., Chaidaki K., Georgopoulou K.-E., Magaliou S., Roussaki Schulze A.V., Bogdanos D.P., Zafiriou E. (2024). Plaque Psoriasis Exacerbation and COVID-19 Vaccination: Assessing the Characteristics of the Flare and the Exposome Parameters. Vaccines.

[5] Marasca C., Ruggiero A., Annunziata M., Fabbrocini G., Megna M. (2020). Face the COVID-19 emergency: Measures applied in an Italian Dermatologic Clinic. J. Eur. Acad. Dermatol. Venereol..

[6] Ruggiero A., Potestio L., Cacciapuoti S., Gallo L., Battista T., Camela E., Fabbrocini G., Megna M. (2022). Tildrakizumab for the treatment of moderate to severe psoriasis: Results from a single center preliminary real-life study. Dermatol. Ther..

[7] Nast A., Smith C., Spuls P.I., Avila Valle G., Bata-Csörgö Z., Boonen H., De Jong E., Garcia-Doval I., Gisondi P., Kaur-Knudsen D. (2020). EuroGuiDerm Guideline on the systemic treatment of Psoriasis vulgaris—Part 1: Treatment and monitoring recommendations. J. Eur. Acad. Dermatol. Venereol..

[8] Greiling T., Young M., Seal M.S., Higham R.C., Armstrong A. (2024). Patient Perspectives on the Prevalence and Burden of Intertriginous Psoriasis: Results from a National Survey of Adults with Psoriasis in the United States. Dermatol. Ther..

[9] Prignano F., Brunasso A.M.G., Fabbrocini G., Argenziano G., Bardazzi F., Borroni R.G., Burlando M., Cagni A.E., Campione E., Cinotti E. (2022). Sharing Patient and Clinician Experiences of Moderate-to-Severe Psoriasis: A Nationwide Italian Survey and Expert Opinion to Explore Barriers Impacting upon Patient Wellbeing. J. Clin. Med..

[10] Galluzzo M., Chiricozzi A., Cinotti E., Brunasso G., Congedo M., Esposito M., Franchi C., Malara G., Narcisi A., Piaserico S. (2022). Tildrakizumab for treatment of moderate to severe psoriasis: An expert opinion of efficacy, safety, and use in special populations. Expert. Opin. Biol. Ther..

[11] European Medicines Agency (2019). Ilumetri (Tildrakizumab): Summary of Product Characteristics. https://www.ema.europa.eu/en/documents/product-information/ilumetri-epar-product-information_en.pdf.

[12] Papp K.A., Reich K., Blauvelt A., Kimball A.B., Gooderham M., Tyring S.K., Sinclair R., Thaci D., Li Q., Cichanowitz N. (2019). Efficacy of tildrakizumab for moderate-to-severe plaque psoriasis: Pooled analysis of three randomized controlled trials at weeks 12 and 28. J. Eur. Acad. Dermatol. Venereol..

[13] Heim J., Vasquez J.G., Schenkel B., Bhatia N. (2023). Real-World Effectiveness and Safety of Tildrakizumab in Patients With Moderate-to-Severe Psoriasis: Week 28 Interim Analysis of a Phase 4 Study. J. Drugs Dermatol..

[14] Ruggiero A., Picone V., Martora F., Fabbrocini G., Megna M. (2022). Guselkumab, Risankizumab, and Tildrakizumab in the Management of Psoriasis: A Review of the Real-World Evidence. Clin. Cosmet. Investig. Dermatol..

[15] Ruggiero A., Fabbrocicni G., Cacciapuoti S., Potestio L., Gallo L., Megna M. (2023). Tildrakizumab for the Treatment of Moderate-to-Severe Psoriasis: Results from 52 Weeks Real-Life Retrospective Study. Clin. Cosmet. Investig. Dermatol..

[16] Amin M., Darji K., No D.J., Wu J.J. (2017). Review of phase III trial data on IL-23 inhibitors tildrakizumab and guselkumab for psoriasis. J. Eur. Acad. Dermatol. Venereol..

[17] Megna M., Ruggiero A., Battista T., Marano L., Cacciapuoti S., Potestio L. (2023). Long-Term Efficacy and Safety of Risankizumab for Moderate to Severe Psoriasis: A 2-Year Real-Life Retrospective Study. J. Clin. Med..

[18] Reich K., Papp K.A., Blauvelt A., Tyring S.K., Sinclair R., Thaçi D., Nograles K., Mehta A., Cichanowitz N., Li Q. (2017). Tildrakizumab versus placebo or etanercept for chronic plaque psoriasis (reSURFACE 1 and reSURFACE 2): Results from two randomised controlled, phase 3 trials. Lancet 2017, 390, 276–288; Erratum in Lancet..

[19] Megna M., Tommasino N., Potestio L., Battista T., Ruggiero A., Noto M., Fabbrocini G., Genco L. (2022). Real-world practice indirect comparison between guselkumab, risankizumab, and tildrakizumab: Results from an Italian 28-week retrospective study. J. Dermatol. Treat..

[20] Campione E., Lambiase S., Gaeta Shumak R., Galluzzo M., Lanna C., Costanza G., Borselli C., Artosi F., Cosio T., Tofani L. (2023). A Real-Life Study on the Use of Tildrakizumab in Psoriatic Patients. Pharmaceuticals.

[21] Narcisi A., Valenti M., Gargiulo L., Ibba L., Amoruso F., Argenziano G., Bardazzi F., Burlando M., Carrera C.G., Damiani G. (2023). Real-life effectiveness of tildrakizumab in chronic plaque psoriasis: A 52-week multicentre retrospective study-IL PSO (Italian landscape psoriasis). J. Eur. Acad. Dermatol. Venereol..

[22] Lim D.S., Bewley A., Oon H.H. (2018). Psychological Profile of Patients with Psoriasis. Ann. Acad. Med. Singap..

[23] da Silva N., Augustin M., Langenbruch A., Mrowietz U., Reich K., Thaçi D., Boehncke W.H., Kirsten N., Danckworth A., Sommer R. (2020). Disease burden and treatment needs of patients with psoriasis in sexually-sensitive and visible body areas: Results from a large-scale survey in routine care. Eur. J. Dermatol..

[24] Jankowiak B., Krajewska-Kułak E., Jakoniuk M., Khvorik D.F. (2023). Stigmatization among Patients with Plaque Psoriasis. J. Clin. Med..

[25] Founta O., Adamzik K., Tobin A.M., Kirby B., Hevey D. (2019). Psychological Distress, Alexithymia and Alcohol Misuse in Patients with Psoriasis: A Cross-Sectional Study. J. Clin. Psychol. Med. Settings.

[26] Griffiths C.E., Richards H.L. (2001). Psychological influences in psoriasis. Clin. Exp. Dermatol..

[27] Poulin Y., Sheth P., Gu Y., Teixeira H.D. (2014). Health-related quality of life worsens disproportionately to objective signs of psoriasis after withdrawal of adalimumab therapy. Dermatol. Ther..

[28] Bożek A., Reich A. (2017). The reliability of three psoriasis assessment tools: Psoriasis area and severity index, body surface area and physician global assessment. Adv. Clin. Exp. Med..

[29] Flytström I., Stenberg B., Svensson Å., Bergbrant I.M. (2012). Patients' visual analogue scale: A useful method for assessing psoriasis severity. Acta Derm. Venereol..

[30] Blauvelt A., Sofen H., Papp K., Gooderham M., Tyring S., Zhao Y., Lowry S., Mendelsohn A., Parno J., Reich K. (2019). Tildrakizumab efficacy and impact on quality of life up to 52 weeks in patients with moderate-to-severe psoriasis: A pooled analysis of two randomized controlled trials. J. Eur. Acad. Dermatol. Venereol..

[31] Augustin M., Sommer R., Daudén E., Laws P., de Jong E., Fabbrocini G., Naldi L., Navarini A., Lambert J., Reguiai Z. (2023). Patient-reported well-being in value-based care using tildrakizumab in a real-world setting: Protocol of a multinational, phase IV, 1-cohort prospective observational study (the POSITIVE study). BMJ Open..

[32] Costanzo A., Llamas-Velasco M., Fabbrocini G., Cuccia A., Rivera-Diaz R., Gaarn Du Jardin K., Kasujee I., Puig L., Carrascosa J.M. (2023). Tildrakizumab improves high burden skin symptoms, impaired sleep and quality of life of moderate-to-severe plaque psoriasis patients in conditions close to clinical practice. J. Eur. Acad. Dermatol. Venereol..

